# *Galleria mellonella* Reveals Niche Differences Between Highly Pathogenic and Closely Related Strains of *Francisella* spp.

**DOI:** 10.3389/fcimb.2018.00188

**Published:** 2018-06-05

**Authors:** Johanna Thelaus, Eva Lundmark, Petter Lindgren, Andreas Sjödin, Mats Forsman

**Affiliations:** Swedish Defence Research Agency (FOI), Umeå, Sweden

**Keywords:** tularemia, *Francisella tularensis*, *Galleria mellonella*, virulence, host specificity, lethality, ecology

## Abstract

*Francisella tularensis*, a highly virulent bacteria that causes the zoonotic disease tularemia, is considered a potential agent of biological warfare and bioterrorism. Although the host range for several species within the *Francisella* is known, little is known about the natural reservoirs of various *Francisella* species. The lack of knowledge regarding the environmental fates of these pathogens greatly reduces the possibilities for microbial risk assessments. The greater wax moth (*Galleria mellonella)* is an insect of the order *Lepidoptera* that has been used as an alternative model to study microbial infection during recent years. The aim of this study was to evaluate *G. mellonella* as a model system for studies of human pathogenic and closely related opportunistic and non-pathogenic strains within the *Francisella* genus. The employed *G. mellonella* larvae model demonstrated differences in lethality between human pathogenic and human non-pathogenic or opportunistic *Francisella* species. The *F. novicida, F. hispaniensis* and *F. philomiragia* strains were significantly more virulent in the *G. mellonella* model than the strains of human pathogens *F. t. holarctica* and *F. t. tularensis*. Our data show that *G. mellonella* is a possible *in vivo model* of insect immunity for studies of both opportunistic and virulent lineages of *Francisella* spp., that produces inverse results regarding lethality in *G. mellonella* and incapacitating disease in humans. The results provide insight into the potential host specificity of *F. tularensis* and closely related members of the same genus, thus increasing our present understanding of *Francisella* spp. ecology.

## Introduction

*Francisella tularensis* is a highly virulent bacteria that causes the zoonotic disease tularemia. This pathogen is considered a potential agent of biological warfare and bioterrorism. As such, it is classified as a Tier 1 select agent (Federal Register, 2012). Three subspecies of *F. tularensis* are commonly accepted: *F. t. holarctica, F. t. tularensis* and *F. t. mediasiatica* (Sjöstedt, 2015). Both *F. tularensis* subspecies *holarctica* and subspecies *tularensis* cause disease in humans (Penn, 2010). However, information on the virulence of *F. t. mediasiatica*, which is primarily found in Central Asia, in humans is limited (Timofeev et al., 2017). Besides *F. tularensis*, the genus *Francisella* also includes opportunistic pathogens that only cause disease in immunocompromised humans (i.e., *F. novicida, F. hispaniensis* and *F. philomiragia*; Larson et al., 1955; Olsufjev et al., 1959; Hollis et al., 1989; Huber et al., 2010; Penn, 2010; Aravena-Román et al., 2015). There is an ongoing debate about whether *F. novicida* should be classified as a separate *Francisella* species or as a subspecies of *F. tularensis* (Busse et al., 2010; Johansson et al., 2010). The facts that *F. novicida* infections are uncommon in humans and that infections have only occurred in immunocompromised individuals or people with underlying health problems reflect that *F. novicida* is indeed an opportunistic pathogen (Kingry and Petersen, 2014). In addition, the genus *Francisella* includes fish pathogens (i.e., *F. noatunensis, F. halioticida*) (Ottem et al., 2008; Brevik et al., 2011), endosymbionts of ticks and ciliates (i.e., *F. persica* and *F. endociliophora*) (Sjödin et al., 2014; Larson et al., 2016), and species with unknown niche preferences, i.e., species isolated from brackish water, air conditioning systems and cooling towers (*F. salina, F. uliginis, F. frigiditurris*, and *Allofrancisella* spp.; Qu et al., 2013, 2016; Challacombe et al., 2017). Thus, representatives of the *Francisella* genus inhabit diverse ecological niches. Rapid developments in high-throughput sequencing technologies during the last years have increased knowledge on the diversity of *Francisella* and its genetic neighbors (Sjödin et al., 2012; Challacombe et al., 2017).

Although previous research has identified the host preferences of several *Francisella* species, little is known about the natural reservoirs of these different *Francisella* species. This lack of knowledge concerning environmental dynamics greatly reduces the possibilities for microbial risk assessments of *Francisella* pathogens However, the increase in genus diversity knowledge is pivotal to discriminating pathogenic from non-pathogenic strains. This could improve environmental bio-surveillance and epidemiological studies that rely on complex sample matrices which are plagued by false positive signals originating from both non-pathogenic *Francisella* species and close genetic neighbors. Still, studies of these newly defined organisms are challenged by the difficulties in culturing these strains in the laboratory.

The greater wax moth (*Galleria mellonella)* is an insect of the order *Lepidoptera* that has been introduced as an alternative model to study microbial infection during recent years (Tsai et al., 2016). When compared to traditional murine models, *G. mellonella* larvae are cheaper and easier to maintain. Furthermore, *G. mellonella* can survive temperatures between 25 and 37°C, which enables researchers to investigate the host-associated replication of human pathogens. Although there are major differences between the immune systems of humans and insects, the innate immune responses of insects and vertebrates share a cellular and a humoral component (Kavanagh and Reeves, 2004; Browne et al., 2013). *G. mellonella* has been used as an infection model to study bacterial and fungal infections, as well as to evaluate the efficacy of antimicrobial substances (Johnson et al., 2015; Tsai et al., 2016; Barnoy et al., 2017; Meir et al., 2018) Additionally, a *G. mellonella* infection model has been established for the *F. t. holarctica* live vaccine strain (LVS) (Aperis et al., 2007).

The aim of this study was to evaluate whether *G. mellonella* can be used as a model system to differentiate human pathogenic strains from closely related opportunistic and non-pathogenic strains within the *Francisella* genus. We show unexpected differences in lethality between human-pathogenic and human non-pathogenic or opportunistic *Francisella* species in the *G. mellonella* larvae model. Thus, the results demonstrate the importance of including non-pathogenic genetic neighbors when evaluating new model systems and suggest niche differences between highly pathogenic and closely related strains of *Francisella*.

## Materials and methods

### Bacterial strains growth conditions

All *Francisella* strains used in this study (Table 1) were cultured on GCII agar containing 1% hemoglobin and 1% IsoVitaleX (World Health Organization, 2007), and complemented with 50 μg/mL ampicillin, 100 μg/mL polymyxin B and 25 μg/mL vancomycin. The cultures were incubated at 37°C (5% CO_2_), except for *F. endociliophora*, which was incubated at 22°C. Laboratory work with FSC200, FSC147, and FSC237 were performed at a Biosafety level 3 (BSL3) laboratory.

**Table 1:** Francisella strains included in the study.

**Strain**	**FSC number**	**References**
*F. t. holarctica* LVS	458	Isolated from vaccine ampoule, no NDBR 101, Pasturella tularensis Vaccine Live, lot no 11, 1964, The National Drug Company, Philadelphia, USA
*F. t. holarctica*	200	Svensson et al., 2012
*F. t. tularensis* SchuS4	237	Larsson et al., 2005
*F. t. mediasiatica*	147	Larsson et al., 2009
*F. novicida*	040	Larson et al., 1955; Rohmer et al., 2007
*F. hispaniensis*	454	Huber et al., 2010
*F. philomiragia*	037	Sjödin et al., 2012
*F. endociliophora*	1006	Sjödin et al., 2014

### Preparation of inocula for infections

Bacteria were suspended in PBS solution to a final count of 1 × 10^9^ bacteria/mL as determined by optical density (OD_600_) measurements, and further diluted to give inocula of 10^8^, 10^6^, and 10^4^ bacteria/mL. The actual infectious dose was confirmed by plating serial dilutions for an analysis of colony forming units (CFU).

### Bacterial growth experiments

Bacteria were suspended in PBS solution to a final count of 10^5^ bacteria/mL. Growth rate experiments were performed on solid agar plates where one μl of bacterial suspension were applicated in eight technical replicates. Growth was monitored at 18, 32, and 48 h and scored when bacterial colonies were visible to the eye.

### Infection of *G. mellonella*

*G. mellonella* larvae in the final larval stage (Vivara/CJ Wildbird Foods Ltd., Shrewsbury, UK) were stored in the dark at 14°C. To assess the differences between bacterial concentrations, 10 μl of the different dilutions described above were injected into the hemocoel of larvae via the last left proleg. After injection, the larvae were incubated in Petri dishes at 37° or 22°C for 9 days. Ten randomly chosen larvae from each group were used to evaluate virulence, and the number of dead larvae was scored every day. Larvae were considered dead when they did not turn after being turned onto their back. All experiments were repeated three times with different batches of larvae. Untreated larvae and larvae injected with PBS were used as controls in all experiments. Upon death or at the end of the experiment, hemolymph was collected from larvae into 1.5 ml Eppendorf tubes for immediate analysis of viable counts or stored at −80°C for confirmative real-time PCR analysis.

### Confirmative culture and real-time PCR

To evaluate the tissue burden of *Francisella* spp. (for FSC200, FSC040, FSC454, and FSC037) in *G. mellonella*, the hemolymph collected from infected larvae was serial diluted and plated onto agar plates as described above. In order to confirm that larval death was caused by *Francisella* sp., all hemolymph samples were screened for the presence of *Francisella* specific DNA using real-time PCR with the primers Fra_ISFtu2_F CCCTGATTTACAAGAAGTC and Fra_ISFtu2_R CTTGGTTATCATCTTTATCATATC and probe Fra_ISFtu2 TGATTCAACAATAGCAAGAGCACAT (for FSC 458, 200, 237, 147, and 040) modified from Versage et al. (2003), and the primers GF1_F AACTGGCTGACCTTCAGCAT and GF1_R GTGGTCGTGGTAAAGCTGGT and GF1 probe CCGATTAGGCTTTCTGCTACTTCACGA (Sjödin, unpublished) (for FSC 037, 454, and 1006). No DNA extraction was performed prior to the PCR, however, all samples were diluted 1/10 in water to avoid inhibition of the PCR process.

Each reaction mixture comprised 1 μl diluted larval hemolymph, 12.5 μl PerfeCTa qPCR ToughMix (Quantabio, Beverly, MA), 0.625 μl of each primer (20 pM), 0.5 μl of probe (5 pM) and MiliQ water to a total volume of 25 μl. An initial denaturation at 98°C for 2 min was followed by 45 cycles of 98°C for 5 s and 60°C for 5 s on an iCycler (Bio-Rad, Hercules, CA). Larval hemolymph samples spiked with *F. t. holarctica* LVS concentrations ranging from 10^4^ to 10^9^ CFU per mL were analyzed in duplicates to test the limit of detection and generate a standard curve for assessing target DNA concentrations. The standard curve was evaluated with FRA_ISFtu2 pimerpair and probe and the GF1 primerpair and probe.

### Statistical analysis

The Kaplan-Meier estimator was used to estimate the survival function of *G. mellonella* larvae. An event was defined as a dead larva meeting the requirement of a positive PCR measurement (ct < 35,97 (GF1) and ct < 29,94 (Fra_ISFtu2), corresponding to 10^5^ cfu/ml. Observations of dead larvae with a negative PCR measurement (ct > 35,97 (GF1) and ct > 29,94 (Fra_ISFtu2)) as well as observations of larvae that had started pupation were considered censored at the time of death and the time of pupation, respectively. The log rank test was used to compare survival functions between groups (i.e., different *Francisella* species). Analyses for each infectious dose at 37°C as well as for the infectious dose of 10^6^ bacteria/mL at 22°C were performed. In addition, we tested the difference in survival functions between strains at 37°C when controlled for the infectious dose effect. The control group was omitted from this analysis due to discrepancy in infectious dose. In each analysis, multiple adjustments were performed using the Tukey-Kramer method, with adjusted *p*-values < 0.05 considered statistically significant. The survival analysis was performed using the PROC LIFETEST in SAS version 9.4 (SAS Institute, Cary, NC). For details see experimental data and sourcecode deposited at figshare (DOI code: 10.6084/m9.figshare.6154919 and DOI data: 10.6084/m9.figshare.6154928).

### Phylogenetic study of *Francisella* species

Sequence files for all publicly available *Francisella* genomes were downloaded from NCBI (Jan 26, 2018). All of the sequences were aligned using progressiveMauve (Darling et al., 2010), and the multiple alignment was transformed to a common coordinate system (Lärkeryd et al., 2014) before it was imported into MEGA7 (Kumar et al., 2016) to construct a neighbor-joining tree. All analysis steps except tree generation were performed using snakemake (Köster and Rahmann, 2012) and Bioconda.

## Results

### *G. mellonella* mortality depends on both *Francisella* species and temperature

**Figure 1:**
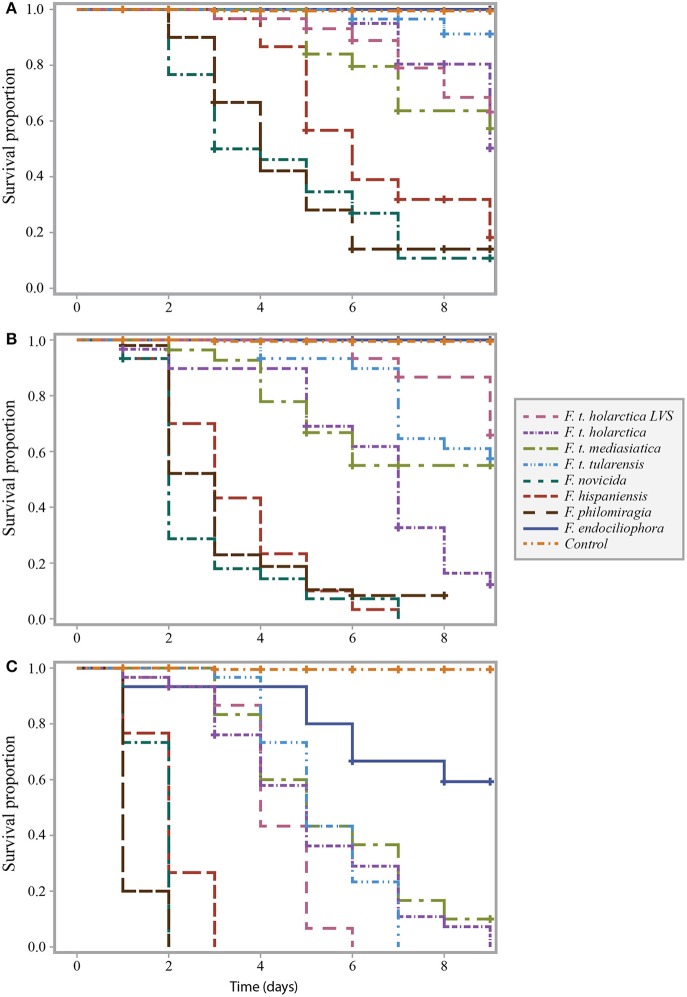
Survival curve comparison of *G. mellonella* infected with *F. t. tularensis, F. t. holarctica, F. t. mediasiatica, F. novicida, F. hispaniensis, F. philomiragia*, and *F. endociliophora* at a temperature of 37°C with an infectious dose of **(A)** 10^4^ bacteria/mL, **(B)** 10^6^ bacteria/mL, and **(C)** 10^8^ bacteria/mL. Experiments were performed in three separate runs with ten larvae in each run for each treatment. Censored observations are indicated with a plus sign (+).

Infection of *Galleria mellonella* with *Francisella* strains resulted in larval death (Figure 1). We infected *G. mellonella* with samples containing 10^4^, 10^6^ or 10^8^ bacteria/mL, resulting in actual infectious doses of 10^2^, 10^4^ and 10^6^ bacteria per larva (low, intermediate and high dose, respectively. see Figures 1A–C).

At a temperature of 37°C, *G. mellonella* larvae were generally more sensitive to the opportunistic *F. novicida, F. hispaniensis*, and *F. philomiragia* strains than the other tested strains (Figure 1). This difference was found to be significant (*p* < 0.001 for all such pairwise combinations) when controlling for infectious dose (Supplementary Table 1, column E). The highest infectious doses of the opportunistic strains resulted in extensive larval death within 3 days while the majority of larvae died within 6–9 days after being injected with the highest infectious doses of the *F. tularensis* strains (*tularensis, holarctica*, and *mediasiatica*). The survival proportion of larvae infected with the highest infectious dose of *F. endociliophora* was approximately 0.6 at the end of the experiment (Figure 1C), while larvae infected with the lowest and intermediate infectious doses were not affected. At an intermediate infectious dose, wild-type *F. t. holarctica* was significantly more virulent than the *F. t. holarctica* LVS (Figure 1B, Supplementary Table 1 column B). This difference was not observed for the lowest or highest infectious dose (Figures 1A,C, Supplementary Table 1 column A and C).

**Figure 2:**
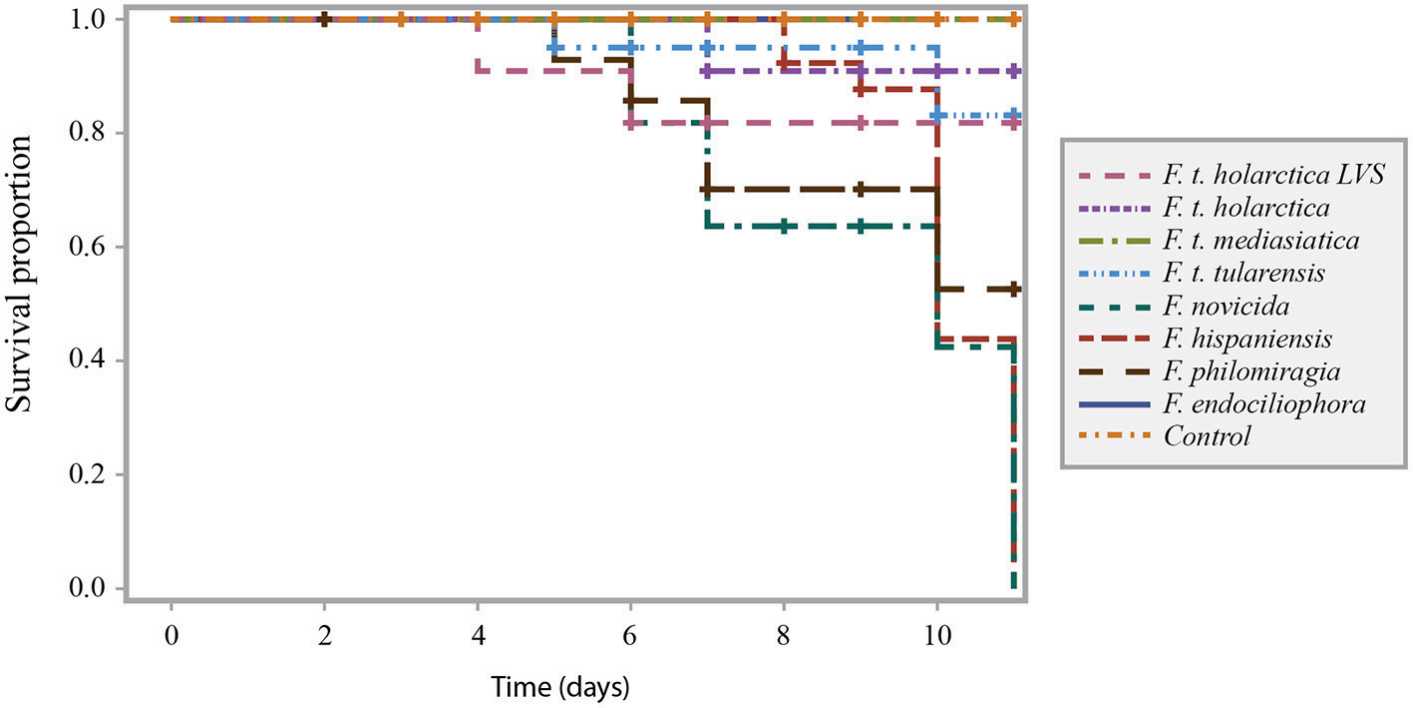
Survival curve comparison of *G. mellonella* infected with *F. t. tularensis, F. t. holarctica, F. t. mediasiatica, F. novicida, F. hispaniensis, F. philomiragia*, and *F. endociliophora* at a temperature of 22°C with an infectious dose of 10^6^ bacteria/mL. Experiments were performed in three separate runs with ten larvae in each run for each treatment. Censored observations are indicated with a plus sign (+).

*Francisella* infection of *G. mellonella* was also temperature-dependent, as higher larval survival proportions were noted when the experiment was performed at 22°C (Figure 2) than when the experiment was performed at 37°C (Figure 1). This trend was noted for all strains at the intermediate infectious dose.

### *Francisella* spp. infect *G. mellonella* with a small infectious dose and grow to high bacterial count

**Figure 3:**
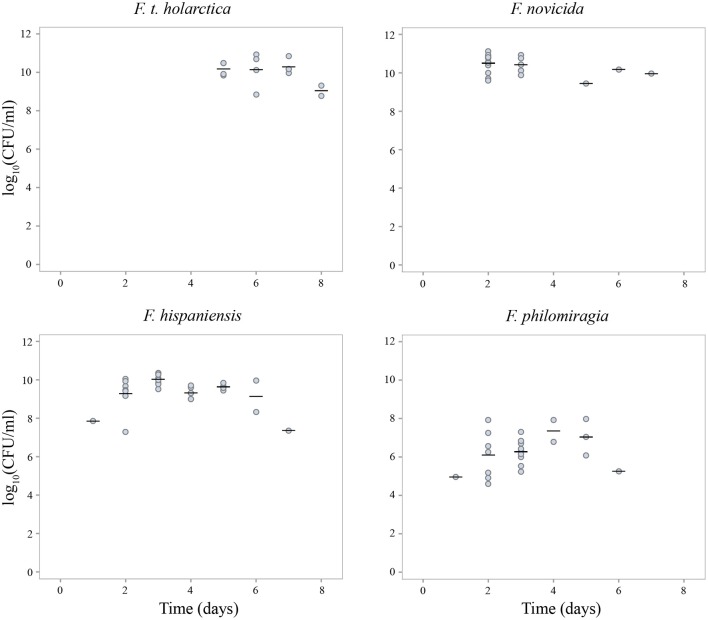
*Francisella* spp. bacterial count in *G. mellonella* hemolymph upon larvae death (*F. t. holarctica, F. novicida, F. hispaniensis, F. philomiragia*). Mean values for each day are indicated by a horizontal line. Experiments were performed in three separate runs with 10 larvae in each run for each treatment. **Larvae producing a negative result in the viable count analysis (*F. t. holarctica* n = 6, *F. novicida* n = 0, *F. hispaniensis* n = 1 and *F. philomiragia* n = 6) were excluded from the figure.

The bacterial load of *Francisella* spp. in *G. mellonella* hemolymph upon larval death reached approximately 10^10^ CFU/mL for *F. t. holarctica, F. novicida* and *F. hispaniensis* (Figure 3). Based on the assumption that each larvae contains 35 μL of hemolymph, the initial 10^4^ bacteria introduced upon infection (intermediate dose) reached 3.5 × 10^8^ bacteria per larva at the time of larval death. However, the bacterial load of *F. philomiragia* in hemolymph upon larval death was noticeably lower than what was observed for other *Francisella* species, not exceeding 10^8^ CFU/mL, which corresponds to 3.5 × 10^6^ bacteria per larva at the time of death (Figure 3).

Taken together, these results suggest that the bacterial load increased by 10^4^ over 2 days in samples infected with *F. novicida* and *F. hispaniensis*, by 10^4^ over 5 days in samples infected with *F. t. holarctica*, and 10^2^ over 2 days in samples infected with *F. philomiragia*. No increase in the bacterial load of *F. endociliophora* in larval hemolymph was observed during the 9-day experiment (data not shown).

### Differences in growth rate between *Francisella* strains on laboratory culture media

Growth of *F. hispaniensis* and *F. philomiragia* was observed after 18h incubation on solid agar media (Supplementary Figure 1). At 32 h colonies of the *F. t. tularensis*, the *F. t. mediasiatica* and the *F. novicida* strains were visible. However, colonies of *F. t. holarctica* and *F. t. holarctica* LVS were not visible until the 48 h time point. *F. endociliophora* was not included in the growth experiment since the strain does not grow at 37°C.

### Host specificity of *francisella* species

**Figure 4:**
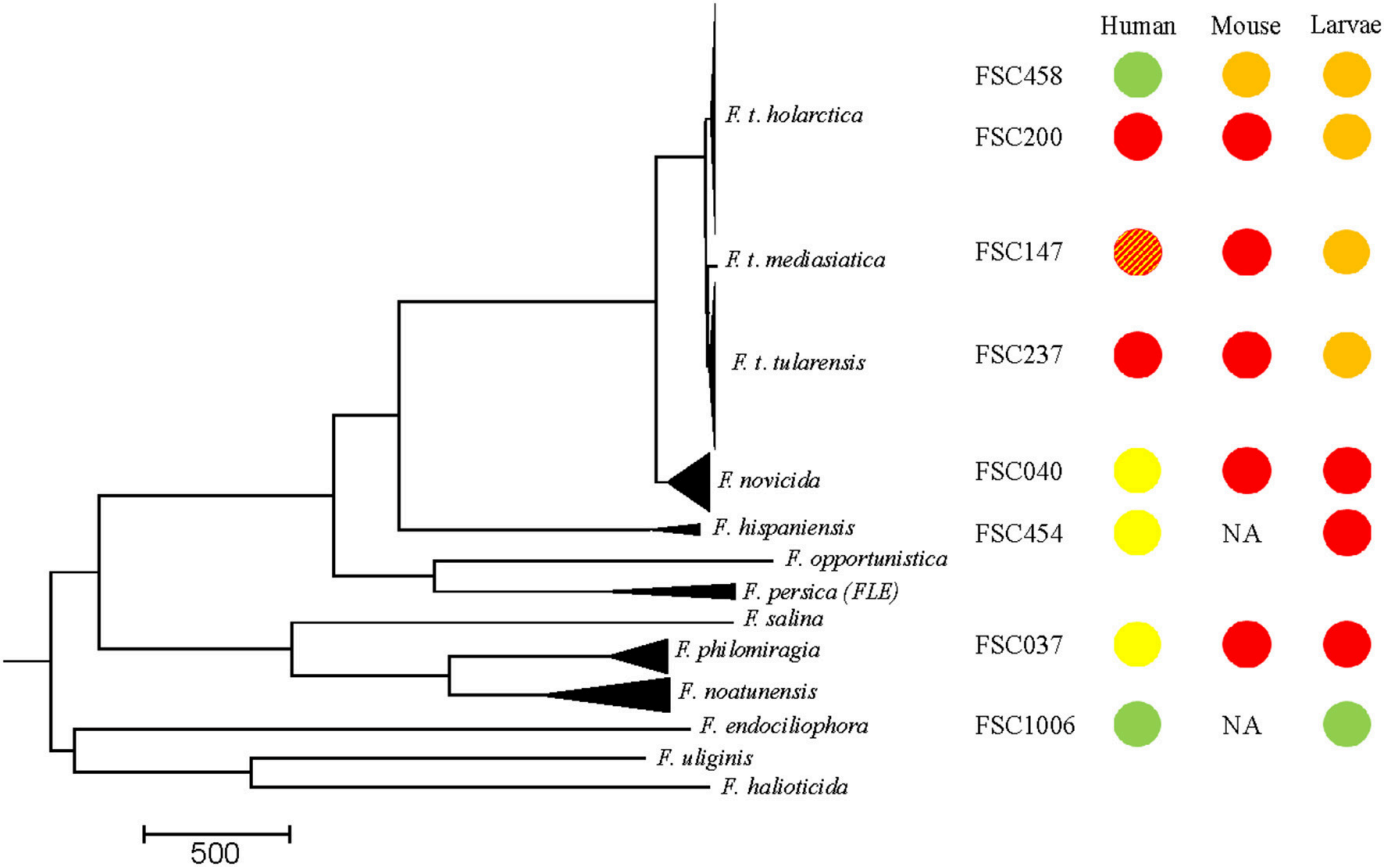
Comparison between data of *Francisella* spp. lethality in *G. mellonella* generated in this study and previously published data on *Francisella* spp. lethality in mice by subcutaneous or intracutaneous inoculation (Bell et al., 1955; Owen et al., 1964; Guryčová, 1998; Kieffer et al., 2003; Salomonsson et al., 2009; Molins et al., 2014; Propst et al., 2016; Timofeev et al., 2017) and severity of disease in humans (Saslaw et al., 1961a,b; Dienst, 1963; Kugeler et al., 2009). Lethality of the different *Francisella* strains in *G. mellonella* (larvae death within 3 days, infectious dose 10^6^ bacteria/mL) and mice (LD_50_ < 10^3^) was scored as high lethality (red), intermediate lethality (larvae death > 3 days, LD_50_ mice > 10^3^) (orange) and non-lethal (green). Severity of disease caused by the *Francisella* strains in humans, was scored as incapacitating disease in humans (red), causing disease only in humans with a compromised immune response or underlying health defects (yellow) and non-virulent (green). There is only limited data on the virulence of *F. t. mediasiatica* in humans (Timofeev et al., 2017) (striped) and data on *F. hispaniensis* and *F. endociliophora* lethality in mice are lacking. The LVS strain is a live attenuated strain that was selected for vaccination in humans but still remains unlicensed for human use (Sandström, 1994). NA, data not available.

The relevance of *G. mellonella* as a model system to differentiate human pathogenic strains from closely related opportunistic and non-pathogenic strains within the *Francisella* genus was evaluated by comparing lethality in *G. mellonella* (this study) with previously published data describing *Francisella* lethality in mice and severity of disease in humans (previously published studies and epidemiological data, respectively, Figure 4). The lethality of different *Francisella* strains in *G. mellonella* (this study) and mice were ranked as either high lethality (red), intermediate lethality (orange) and non-lethal (green). Similarly, the severity of disease caused by *Francisella* strains in humans was scored as incapacitating disease in humans (red), causing disease only in humans with a compromised immune response or underlying health defects (yellow), and non-virulent (green) (Figure 4). Our data show an inverse relationship, as the opportunistic pathogens in humans (*F. novicida, F. hispaniensis*, and *F. philomiragia*) are highly lethal in *G. mellonella* (Figure 4), while the human pathogens (*F. t. tularensis* and *F. t. holarctica*) show intermediate lethality in *G. mellonella*. *F. endociliophora* does not cause disease in either humans or *G. mellonella*.

## Discussion

Our *G. mellonella* model identified differences in lethality between *Francisella* species that do not correspond to severity of disease in humans as shown by available epidemiological and experimental data. The *F. novicida, F. hispaniensis* and *F. philomiragia* strains were significantly more lethal in the *G. mellonella* model than the *F. t. holarctica* and *F. t. tularensis* strains that represent human pathogens. The results provide insight into the potential host specificity of *F. tularensis* and reflect different adaptation to an insect host (*G. mellonella*) between *F. tularensis* and its near-neigbours. Arthropode borne transmission of human pathogenic *F. tularensis* species is widely documented and vectors considered significant for the transmission of *F. tularensis* to humans are hard tick, mosquito, deer-fly, and horse-fly (Petersen et al., 2009; Pilo, 2018). Our findings suggest that although phylogenetically close to the human pathogenic strains, the *F. novicida, F. hispaniensis*, and *F. philomiragia* are more adapted for rapid growth in the insect host model.

The strains investigated in this study were separated into three lineages (Figure 4), i.e., the *F. tularensis* lineage (*F. t. tularensis, F. t. mediasiatica, F. t. holarctica*, and *F. t. holarctica* LVS), the more diverse group comprising animal and opportunistic human pathogens associated with water environments (*F. novicida, F. hispaniensis*, and *F. philomiragia*) and a lineage containing only *F. endociliophora*, a recently identified endosymbiont of the marine ciliate *Euplotes raikovi*. We can only provide speculative explanations for the inverse result of the first and second *Francisella* lineages in humans and *G. mellonella* as knowledge of *Francisella* spp. virulence mechanisms has mainly been restricted to studies of *F. t. holarctica, F. t. tularensis* and *F. novicida*. Strains in the first lineage (*F. tularensis*), are characterized by selective genome reduction, which has resulted in deletions of metabolic pathway components (i.e., genes involved in amino acid biosynthesis) as a specialized intracellular parasite can acquire the nutrients required for growth and replication upon infecting a host (Larsson et al., 2005, 2009). In comparison, the second lineage of opportunistic strains and specifically, the extensively studied species *F. novicida*, are more metabolically versatile, less fastidious and show higher growth rates (Owen et al., 1964; Rohmer et al., 2007). In addition *F. novicida* elicits a different immune response in the mammalian host than *F. t. tularensis* and *F. t. holarctica*, causing disease only in immunocompromised persons (Kingry and Petersen, 2014). Our results corroborate previous studies that have shown vigorous growth of *F. novicida* in hemocytes (Santic et al., 2009). Studies of *F. novicida* within arthropod cells identified similar molecular mechanisms of pathogenesis, i.e., intracellular trafficking, as in mammalian cells (Ozanic et al., 2015), but the species nevertheless utilizes different virulence factors for proliferation in mammal and arthropod cells (Read et al., 2008; Santic et al., 2009; Åhlund et al., 2010).

A distinguishing feature of *F. tularensis* is its ability to evade the host immune response in mammals (Sjöstedt, 2006). *F. tularensis* maintain a low immunological profile early during the infection process by evading immune system surveillance, and only later replicate within the relatively protective environment of the host cell cytoplasm (Jones et al., 2014; Steiner et al., 2014). One important factor in *F. tularensis* immune evasion is an atypical lipopolysaccharide (LPS) that exhibits very low endotoxicity and stimulation of inflammatory pathways (Hajjar et al., 2006). In contrast, *F. novicida* exhibits a structurally and antigenically different LPS which elicits an inflammatory response (Jones et al., 2014). Since the growth of *Francisella* in hemocytes and macrophages shares many similarities, it is possible that the pathways of the *G. mellonella* humoral immune response (Browne et al., 2013; Tsai et al., 2016) differ from those in humans regarding early detection of *Francisella* species, and that the difference in lethality observed in our experiments (Figure 3) rather reflects growth rate differences (Supplementary Figure 1), with the metabolically versatile opportunistic *Francisella* strains having an advantage in *G. mellonella*.

The growth of *F. philomiragia* was most detrimental to the host (*G. mellonella*). The larvae succumbed at a lower bacterial load than what was observed for the other strains tested in this study. A more detrimental effect from growth of *F. philomiragia*, in comparison with *F. tularensis* ssp. and *F. novicida*, was also reported in the ciliate *T. pyriformis* (Thelaus et al., 2009). Virulence factors in *F. philomiragia* have been less studied, but based on the genome sequence, this species is likely to express similar proteins as *F. tularensis* and *F. novicida* (Zeytun et al., 2012). Previous studies have shown that *F. philomiragia* can infect and grow in macrophages, lung, and liver cells as well as in *T. pyriformis* and *G. mellonella* (Thelaus et al., 2009; Propst et al., 2016). This suggests that *F. philomiragia*, like *F. tularensis* ssp. and *F. novicida*, can infect and proliferate in many hosts and cell types. Although *F. philomiragia* stands out as the most aggressive strain in our experiments, all of the opportunistic strains resulted in *G. mellonella* death significantly faster than the three *F. tularensis* strains tested.

Finally, the third lineage, which includes only the endosymbiont *F. endociliophora*, represents a specific niche that is restricted to growth in a ciliate host and at lower temperature compared to the other strains tested (Sjödin et al., 2014). In contrast to the *F. tularensis* and opportunistic strains, no growth of *F. endociliophora* in *G. mellonella* was recorded. This is in line with the high degree of host restriction that is characteristic to primary endosymbionts, as well as the adaptation to host growth rates, which ensures that bacteria are transferred during host cell division (Fokin, 2004). Interestingly, *F. endociliophora* branch early in the phylogenetic tree of the *Francisella* genus (Figure 4) but display a more specialized lifestyle than the more recently branching *Francisella* species that are characterized by the ability to replicate in a broad range of hosts.

We only detected a significant difference in the lethality between wild-type *F. t. holarctica* and the live vaccine strain (LVS) at the intermediate infectious dose. No difference in lethality between these two strains was found in the *G. mellonella* system when the results were corrected for dose (Supplementary Table 1, column E). It could be speculated that the doses might have affected this result, with the highest dose too high to allow differentiation of the two strains, and the lower dose may have revealed differences if the study duration was extended. This should be considered in any further experiments.

Larval death following infection was less pronounced at 22°C than at 37°C for all of the tested strains, and the difference between the *F. tularensis* and opportunistic strains was less pronounced at 22°C. It is possible that prolonged incubation would have revealed lethality differences between strains. However, *G. mellonella* larvae have been reported to survive incubation at 37°C but prolonged incubation at this temperature may induce heat stress that renders the larvae more sensitive to infection. Thus, it is plausible that the effect of temperature on larval survival can be explained by incubation at 37°C representing a temperature that is close to the upper limit for *G. mellonella* larvae.

In 2007, Aperis et al. introduced *G. mellonella* as a model host for studies of LVS and antibacterial agent efficacy (Aperis et al., 2007). In addition, Propst and colleagues compared different model systems, including *G. mellonella* to clarify the use of *F. philomiragia* as a model for studies of *Francisella* (Propst et al., 2016). These previous studies, along with our data, show that *Francisella* spp. strains multiply in, and eventually kill, *G. mellonella* larvae, which proves their utility as a model for virulence studies. This in line with numerous studies of *G. mellonella* as a model for bacterial virulence, the majority of which present good correspondence regarding bacterial pathogenicity in humans and *G. mellonella* (Sprynski et al., 2014; Tsai et al., 2016). Our data clearly show that the strains that cause incapacitating disease in humans are not as efficient in replicating in the larval host model as the *F. novicida, F. hispaniensis*, and *F. philomiragia* strains. Thus, *Francisella* spp. lethality in *G. mellonella* is not reflective of severity of disease in humans. This in line with a study of pathogenic *Escherichia coli* (causing urosepsis) in *G. mellonella* that conclude that the larval lethality model cannot be a substitute for the murine sepsis model (Johnson et al., 2015). On the other hand, Mukherjee et al. report on *G. mellonella* as a model system that reflects severity of disease of human pathogenic and non pathogenic environmental strains of *Listeria* spp. (Mukherjee et al., 2010). In addition, pathogenic *Yersinia enterocolitica* cause lethality in *G. mellonella* but other environmental strains of *Yersinia* showed high variability in insecticidal potential (Fuchs et al., 2008). Although, the innate immune responses of insects and vertebrates share a cellular and a humoral component, the evolutionary distance between insect and murine models makes it clear that many host-specific phenomena are likely to exist (Scully and Bidochka, 2006). The *G. mellonella* model may or may not result in a bias toward genes relevant for evading insect immunity. This will ultimately depend on the evolutionary history and ecological niche of the bacterial pathogen studied. A possible adaptation of the bacteria to an insect host would be reflected in the *G. mellonella* model system.

A limitation of *G. mellonella* as a model is the lack of standardization that results in unknown variation between sources that may interfere with the experimental outcome. This variation limit the possibility to directly compare data from studies performed at different laboratories. The data presented here were generated using three different batches of *G. mellonella* from the same source (Vivaria). In addition, we performed control experiments with two strains (*F. t. holarctica* LVS and *F. novicida*) using larvae from an alternative source (TruLarv^TM^ BioSystems Technology Ltd, UK). Infection of larvae from both sources with the two strains produced similar lethality results, but larval death in the TruLarv^TM^ system occurred 2 days later than in the Vivaria system (data not shown). This result highlights the need for comparative studies in the search for consistent model systems.

The *G. mellonella* data presented here illustrate possible niche differences of *Francisella* species and contribute to the effort to understand pathogenic *F. tularensis* ssp. in the context of near genetic neighbors. Larvae or insects may play a role as hosts for the replication of opportunistic human pathogens often associated with environmental samples and water. Accordingly, *G. mellonella* is a possible *in vivo* model of insect immunity that can be used for studies of both opportunistic and virulent lineages of *Francisella* spp., that produces inverse results regarding lethality in *G. mellonella* and incapacitating disease in humans. Further studies are needed in order to detangle the specific mechanism that render *Francisella* spp. strains lethal to *G. mellonella*.

## Author contributions

JT designed the study, performed experiments and drafted the manuscript. EL performed experiments and contributed to the design of the study. PL contributed to the design of the study and performed the statistical analyses. AS performed the phylogenetic analysis. MF contributed to the design of the study and writing of the manuscript. All authors contributed to manuscript revision, as well as read and approved the submitted version.

### Conflict of interest statement

The authors declare that the research was conducted in the absence of any commercial or financial relationships that could be construed as a potential conflict of interest.

## Supplementary material

Supplementary Figure 1 Growt rate of Francisella spp. strains (*F. t. tularensis, F. t. holarctica, F. t. mediasiatica, F. t. holarctica LVS, F. hispaniensis, F. philomiragia*, and *F. novicida*) at 37°C on laboratory media. Bacteria was seeded onto agar plates in eight technical replicates and growth was monitored at 0, 18, 32, and 48 h. Growt was scored when bacterial colonies were visible to the eye.

Supplementary Table 1 *P*-values obtained from the log rank test for comparison of strains concerning survival estimates at (A) 37°C and infectious dose of 10^4^ bacteria/mL, (B) 37°C and infectious dose of 10^6^ bacteria/mL, (C) 37°C and infectious dose of 10^8^ bacteria/mL, (D) 22°C and infectious dose of 10^6^ bacteria/mL and (E) 37°C controlled for infectious dose. The *p*-values are adjusted for multiple testing according to the Tukey-Kramer method.
