# Supplementary figures and images for: Galleria mellonella Reveals Niche Differences Between Highly Pathogenic and Closely Related Strains of Francisella spp
Source: Front Cell Infect Microbiol. 2018 Jun 5;8:188. doi: 10.3389/fcimb.2018.00188 (PMC5996057; doi:10.3389/fcimb.2018.00188)

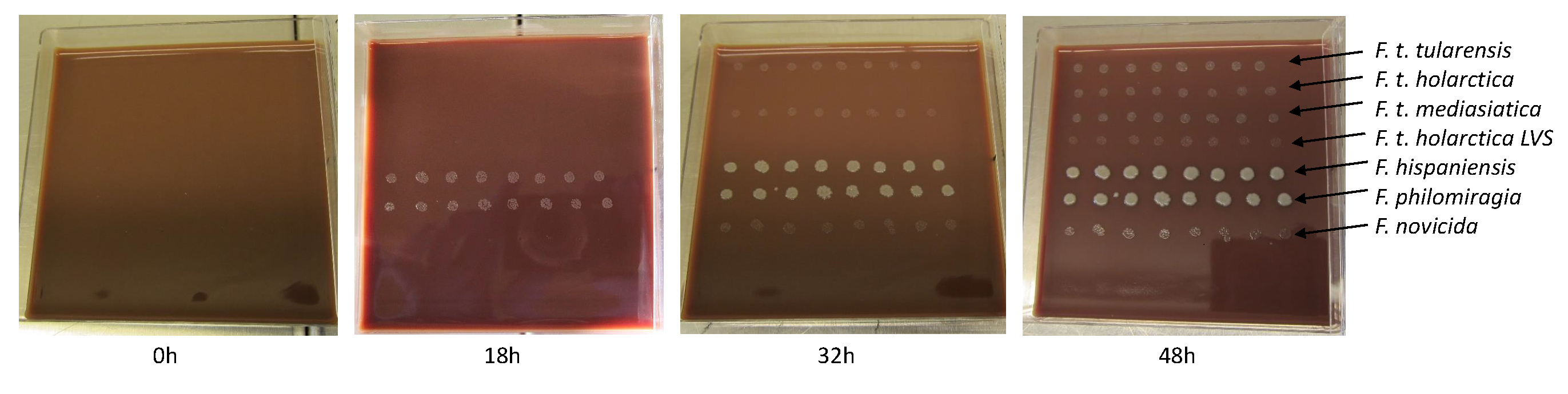

Supplement: Supplementary Figure 1 — Growt rate of Francisella spp. strains (F. t. tularensis, F. t. holarctica, F. t. mediasiatica, F. t. holarctica LVS, F. hispaniensis, F. philomiragia, and F. novicida) at 37°C on laboratory media. Bacteria was seeded onto agar plates in eight technical replicates and growth was monitored at 0, 18, 32, and 48 h. Growt was scored when bacterial colonies were visible to the eye. [file Image_1.TIFF]
