# Supplementary material for: Galleria mellonella Reveals Niche Differences Between Highly Pathogenic and Closely Related Strains of Francisella spp
Source: Front Cell Infect Microbiol. 2018 Jun 5;8:188. doi: 10.3389/fcimb.2018.00188 (PMC5996057; doi:10.3389/fcimb.2018.00188)
Supplement: Supplementary Table 1 — P-values obtained from the log rank test for comparison of strains concerning survival estimates at (A) 37°C and infectious dose of 104 bacteria/mL, (B) 37°C and infectious dose of 106 bacteria/mL, (C) 37°C and infectious dose of 108 bacteria/mL, (D) 22°C and infectious dose of 106 bacteria/mL and (E) 37°C controlled for infectious dose. The p-values are adjusted for multiple testing according to the Tukey-Kramer method. [file Table_1.DOCX]

Supplementary Table 1. P-values obtained from the log rank test for comparison of strains concerning survival estimates at (A) 37°C and infectious dose of 10^4^ bacteria/mL, (B) 37°C and infectious dose of 10^6^ bacteria/mL, (C) 37°C and infectious dose of 10^8^ bacteria/mL, (D) 22°C and infectious dose of 10^6^ bacteria/mL and (E) 37°C controlled for infectious dose. The p-values are adjusted for multiple testing according to the Tukey-Kramer method

| ***Strain1*** | ***Strain2*** | ***p-value*** | | | |  |
| --- | --- | --- | --- | --- | --- | --- |
|  |  | *A* | *B* | *C* | *D* | *E* |
| *Control* | *F. endociliophora* | <.001 | <.001 | <.001 | 0.010 | NA |
| *Control* | *F. hispaniensis* | <.001 | <.001 | <.001 | <.001 | NA |
| *Control* | *F. novicida* | <.001 | <.001 | <.001 | <.001 | NA |
| *Control* | *F. philomiragia* | <.001 | <.001 | <.001 | <.001 | NA |
| *Control* | *F. t. holarctica* | <.001 | <.001 | <.001 | 0.032 | NA |
| *Control* | *F. t. holarctica LVS* | <.001 | <.001 | <.001 | <.001 | NA |
| *Control* | *F. t. mediasiatica* | <.001 | <.001 | <.001 | 0.025 | NA |
| *Control* | *F. t. tularensis* | <.001 | <.001 | <.001 | 0.052 | NA |
| *F. endociliophora* | *F. hispaniensis* | <.001 | <.001 | <.001 | 0.499 | <.001 |
| *F. endociliophora* | *F. novicida* | <.001 | <.001 | <.001 | <.001 | <.001 |
| *F. endociliophora* | *F. philomiragia* | <.001 | <.001 | <.001 | 0.099 | <.001 |
| *F. endociliophora* | *F. t. holarctica* | 0.997 | <.001 | 0.005 | 1.000 | <.001 |
| *F. endociliophora* | *F. t. holarctica LVS* | 0.993 | 1.000 | <.001 | 0.763 | 0.021 |
| *F. endociliophora* | *F. t. mediasiatica* | 0.814 | 0.941 | 0.057 | 1.000 | 0.022 |
| *F. endociliophora* | *F. t. tularensis* | 0.961 | 1.000 | 0.008 | 1.000 | 0.521 |
| *F. hispaniensis* | *F. novicida* | 0.946 | 1.000 | 1.000 | 0.775 | 0.912 |
| *F. hispaniensis* | *F. philomiragia* | 0.845 | 0.106 | 0.995 | 1.000 | 0.116 |
| *F. hispaniensis* | *F. t. holarctica* | <.001 | 0.128 | 0.257 | 0.700 | <.001 |
| *F. hispaniensis* | *F. t. holarctica LVS* | <.001 | <.001 | 0.778 | 0.994 | <.001 |
| *F. hispaniensis* | *F. t. mediasiatica* | 0.003 | <.001 | 0.070 | 0.445 | <.001 |
| *F. hispaniensis* | *F. t. tularensis* | <.001 | <.001 | 0.322 | 0.828 | <.001 |
| *F. novicida* | *F. philomiragia* | 1.000 | 0.051 | 0.999 | 0.899 | 0.727 |
| *F. novicida* | *F. t. holarctica* | <.001 | 0.108 | 0.166 | 0.004 | <.001 |
| *F. novicida* | *F. t. holarctica LVS* | <.001 | <.001 | 0.653 | 0.033 | <.001 |
| *F. novicida* | *F. t. mediasiatica* | <.001 | <.001 | 0.038 | <.001 | <.001 |
| *F. novicida* | *F. t. tularensis* | <.001 | <.001 | 0.221 | 0.023 | <.001 |
| *F. philomiragia* | *F. t. holarctica* | <.001 | <.001 | 0.030 | 0.310 | <.001 |
| *F. philomiragia* | *F. t. holarctica LVS* | <.001 | <.001 | 0.258 | 0.861 | <.001 |
| *F. philomiragia* | *F. t. mediasiatica* | <.001 | <.001 | 0.005 | 0.099 | <.001 |
| *F. philomiragia* | *F. t. tularensis* | <.001 | <.001 | 0.050 | 0.515 | <.001 |
| *F. t. holarctica* | *F. t. holarctica LVS* | 1.000 | 0.006 | 0.998 | 0.933 | 0.865 |
| *F. t. holarctica* | *F. t. mediasiatica* | 0.999 | 0.219 | 1.000 | 1.000 | 0.864 |
| *F. t. holarctica* | *F. t. tularensis* | 0.778 | 0.052 | 1.000 | 1.000 | 0.167 |
| *F. t. holarctica LVS* | *F. t. mediasiatica* | 1.000 | 0.881 | 0.941 | 0.706 | 1.000 |
| *F. t. holarctica LVS* | *F. t. tularensis* | 0.744 | 0.999 | 0.999 | 0.980 | 0.932 |
| *F. t. mediasiatica* | *F. t. tularensis* | 0.363 | 0.998 | 1.000 | 1.000 | 0.929 |
